# A utility of air–liquid interface cultured airway epithelium for the development of antiviral agents: translation beyond biology

**DOI:** 10.3389/fcimb.2026.1892573

**Published:** 2026-07-06

**Authors:** Kazuhiro Ito

**Affiliations:** National Heart and Lung Institute, Imperial College London, London, United Kingdom

**Keywords:** air-liquid interface, antiviral drug, controlled human infection model (CHIM), epithelium, respiratory syncytial virus, respiratory virus, translation

## Abstract

The development of antiviral therapies for respiratory viruses continues to be a major unmet clinical need. One of the key challenges in antiviral drug discovery is the identification of preclinical systems that faithfully reproduce essential features of the human airway while retaining clinical relevance. Primary human airway epithelial cells cultured under air–liquid interface (ALI) conditions provide a differentiated and polarized epithelial model with functional cilia, mucus production, barrier formation, and innate antiviral responses. Importantly, patterns of viral replication and host responses observed in ALI cultures often parallel findings from human infection studies and controlled viral challenge models. Beyond mechanistic studies of virus–epithelium interactions, ALI platforms are increasingly applied to translational decision-making, including dose prediction, biomarker assessment, alignment of preclinical and clinical endpoints, and estimation of clinical trial design parameters. Clinical experience with antiviral agents such as an RSV polymerase inhibitor (PC786) and a narrow-spectrum kinase inhibitor (RV568) further supports the translational utility of these systems. This review summarises the biological rationale for ALI airway models and highlights their expanding role in bridging mechanistic research with early clinical development.

## 1 Introduction

Respiratory viruses such as influenza virus, pandemic coronavirus, respiratory syncytial virus (RSV), and human rhinovirus (HRV) are leading causes of acute respiratory illness and exacerbations of chronic airway diseases (Kesson, 2007; Wedzicha and Seemungal, 2007). Despite extensive advances in molecular virology and immunology, translation of antiviral efficacy from preclinical models to humans remains inconsistent. Traditional screening platforms frequently rely on immortalised cancer-derived cell lines that lack epithelial differentiation, mucociliary architecture, and physiologic innate immune signaling. These limitations can lead to overestimation of antiviral potency and inaccurate dose prediction.

The human airway epithelium is a complex, multicellular barrier composed of ciliated, goblet, club, and basal cells arranged in a pseudostratified structure. Beyond acting as a physical barrier, it performs coordinated mucociliary clearance and produces cytokines, chemokines, and interferons that shape antiviral immunity (Knight and Holgate, 2003; Vareille et al., 2011). Preserving this structural and functional complexity is essential for physiologically meaningful modelling of infection and therapeutic intervention.

Air–liquid interface (ALI) culture of primary human airway epithelial cells has emerged as a powerful system that recapitulates these features (Pezzulo et al., 2011). Importantly, the translational utility of ALI models extends beyond biological observation to prediction of clinical outcomes, dose selection, and biomarker development. This review synthesizes current knowledge on ALI systems and highlights their expanding role in antiviral drug development.

## 2 Biological fidelity of the ALI model

In ALI culture, primary bronchial or nasal epithelial cells are grown on permeable inserts with media supplied basolaterally while the apical surface is exposed to air. This configuration promotes differentiation into a polarized, pseudostratified epithelium with functional cilia, mucus production, and tight junction formation. Receptor distribution, epithelial barrier integrity, and innate immune responsiveness closely resemble in vivo airway tissue (Pezzulo et al., 2011). These features allow assessment of virus-induced epithelial dysfunction and restoration of mucociliary activity following treatment, endpoints that cannot be adequately reproduced in conventional submerged cell lines (**Table 1**). Since impaired mucociliary clearance contributes to disease severity and viral persistence in several respiratory infections, these measurements may provide clinically relevant pharmacodynamic biomarkers, especially in virus challenge in humans (CHIM: controlled human Infection Model). Viral entry receptors are expressed in appropriate cellular compartments, and interferon-mediated antiviral pathways are intact. Consequently, infection kinetics for influenza, RSV, and HRV in ALI cultures mirror the natural temporal course observed in humans, including peak viral replication and subsequent decline (Tapparel et al., 2013; Boda et al., 2018; Brookes et al., 2018; Lopes et al., 2025).

**Table 1:** Comparison of the translational relevance of submerged culture and ALI airway epithelium readouts to human viral challenge studies (CHIM model).

Readout in CHIM	ALI epithelium	Submerge culture of an immortalised cell line
Full virus kinetics from infection to clearance (days)	✓	x
Peak virus load	✓	✓
AUC viral load	✓	x
Viral load reduction slope	✓	x
Time-to-be virus non-detectable	✓	x
Biomarkers	✓	✓
Mucociliary clearance	✓	✓
Mutation induction	✓ (clinically relevant)	✓ (forced mutation over several passages)
Immune cell response, antibody titre	x	x
PK (exposure-clearance)	✓x	✓x
Donor-donor variability	✓	x
Symptom score, lung function	x	x

x: not applicable.

Furthermore, ALI models generate cytokine and chemokine profiles similar to those measured in nasal washes and bronchoalveolar samples from infected individuals. Induction of CXCL10 (IP-10), CCL5 (RANTES), IL-8, and interferon-stimulated genes such as Myxovirus resistant protein-1 (MX1) parallels clinical biomarker patterns, reinforcing the physiological relevance of this system.

## 3 ALI systems in antiviral drug evaluation

### 3.1 Concordance with clinical efficacy

A growing body of evidence demonstrates that antiviral activity observed in ALI cultures predicts outcomes in human challenge studies and early-phase trials. The inhaled RSV polymerase inhibitor PC786 showed potent inhibition of RSV replication in differentiated airway epithelium prior to clinical validation in a human RSV challenge model (Coates et al., 2017; Brookes et al., 2018; DeVincenzo et al., 2022). Nirmatrelvir has also proved to be effective in ALI culture in vitro and in the clinic (Toselli et al., 2026).

The narrow-spectrum kinase inhibitor RV568 provides another example in which epithelial model data informed progression into clinical studies (e.g., NCT01230645) (Cass et al., 2013; Charron et al., 2017). During COVID pandemic, Chloroquine was discovered as a potential anti-viral agent in cancer cell lines, which was confirmed to be clinically less beneficial in clinical trials (Manivannan et al., 2021). Lately, in vitro study with human epithelium confirmed the negative effects of chloroquine (Maisonnasse et al., 2020). This is another example of the translational outcome of ALI culture system. However, the clinical dose for aerolisation of chloroquine was predicted using ALI culture data recently (Kolli et al., 2022).

These examples support the concept that ALI models offer greater predictive concordance than undifferentiated cell lines, particularly for inhaled antiviral strategies targeting the respiratory epithelium.

## 4 Translational applications beyond proof-of-concept

### 4.1 Dose prediction and pharmacodynamic modelling

Conventional antiviral dose selection frequently relies on IC_90_ values derived from immortalized cell lines (monolayer culture). Clinical doses are often estimated as multiples of these in vitro potency values, typically four- to ten-fold higher than IC90/95 (Mackman et al., 2015; Toselli et al., 2026). However, such approaches neglect epithelial differentiation, mucus barriers, and polarized drug exposure.

ALI cultures provide a more physiologically constrained environment. Drug penetration must occur across mucus and tight junctions, and viral replication occurs within differentiated ciliated cells rather than transformed monolayers. Notably, effective concentrations derived from ALI models often align more closely with in vivo efficacy than values obtained from cancer cell lines. This alignment enables more rational selection of inhaled or topical dosing regimens and reduces the risk of under- or over-estimation of therapeutic requirements.

For example, PC786, a small-molecule antiviral compound targeting the RNA-dependent RNA polymerase of respiratory syncytial virus (RSV), was originally tested in ALI culture system and translated to a human viral challenge trial (Coates et al., 2017; Brookes et al., 2018; Mirabelli et al., 2018; DeVincenzo et al., 2022). PC786 is a very potent compound with an IC_90_ of 0.451 ng/mL in RSV-induced CPE assay in Hep2 cells (Coates et al., 2017), and x4 IC_90_ is estimated to be 0.001mg of human estimated dose (HED) using the PK-PD model algorithm developed in-house at Pulmocide Ltd. (Dose justification in clinical protocol) (Coates et al., 2017; DeVincenzo et al., 2022). In contrast, PC786 is required more to inhibit RSV virus burden in ALI airway epithelium model (IC_90_ AUC = 799 ng/mL) (Brookes et al., 2018), therefore, x4 IC_90_ is equivalent to 2 mg HED. The value is similar to the HED generated in the cotton rat *in vivo* model (8 mg HED based on ID_90_ of 712 μg/kg) (Coates et al., 2017). By integrating these preclinical datasets, in silico analyses predicted that an HED would be approximately 5–10 mg. Based on this prediction, a human RSV challenge study was conducted in infected volunteers using a regimen of PC786 administered at 5 mg twice daily for five days. As a result, approximately 70% inhibition of viral load (DeVincenzo et al., 2022). If the anti-viral IC_90_ value from classic submerged monolayer culture was used, the clinical trial would have failed.

### 4.2 Clinical endpoint simulation

A major limitation of conventional in vitro antiviral screening systems with submerged culture of a cell line is that many biological endpoints do not directly correspond to clinical efficacy outcomes. Controlled Human Infection Models (CHIMs) provide one of the most informative early clinical settings for antiviral development because they generate detailed temporal datasets describing viral replication, symptom progression, host responses, and treatment effects under controlled conditions. Consequently, preclinical models that reproduce CHIM-derived endpoints are particularly valuable for translational drug development (**Table 1**).

The main advantage of ALI system is a longer shelf life, as after fully differentiated, cells don’t grow further, unlike monolayer culture, which is overconfluent within 7 days, in contrast to monolayer culture with immortalized cells, which keep growing. Therefore, ALI cultures are capable of modelling longitudinal viral kinetics, including viral expansion, peak replication, and subsequent decline in viral burden (**Table 1**), but do not fully recapitulate immune-mediated viral clearance observed in vivo (see the limitation section).

Thus, ALI systems allow quantification of endpoints directly translatable to human challenge trials (CHIM: controlled human Infection Model). These include time to non-detectable virus, peak viral load, and slope of viral decay (Brookes et al., 2018). Because viral replication follows a defined temporal pattern in differentiated epithelium, modelling of viral kinetics in ALI cultures can simulate early-phase clinical readouts. For example, PC786, anti-RSV agent, achieved 70% inhibition of live RSV viral load in the RSV human challenge, while a highly comparable antiviral effect was observed in ALI culture in vitro, showing 69% inhibition of viral AUC (Brookes et al., 2018; DeVincenzo et al., 2022). As summarized in **Table 1**, differentiated primary airway epithelial cultures maintained under ALI conditions demonstrate a substantially greater overlap with CHIM endpoints than conventional submerged cultures of immortalized cell lines.This approach would facilitate go/no-go decision-making before expensive human studies.

In addition, resistance monitoring represents another area where ALI cultures show translational advantages. Antiviral resistance mutations emerging in ALI cultures are generated under physiologically relevant replication conditions within differentiated human airway epithelium. In contrast, resistance studies in immortalised cell lines frequently require extensive serial passaging under high drug pressure, which may select mutations of uncertain clinical significance. The emergence of resistance-associated variants within ALI systems may therefore better reflect evolutionary pressures encountered during human infection (**Table 1**).

### 4.3 Biomarker identification

The ALI platform reproduces epithelial inflammatory responses observed in patients. Measurement of CXCL10, CCL5, IL-8, interferon-stimulated genes, epithelial damage marker dsDNA, and epithelial injury markers in apical washes or basolateral supernatants enables identification of pharmacodynamic biomarkers as the induction of these biomarkers was confirmed in a human RSV challenge clinical study (Brookes et al., 2018; DeVincenzo et al., 2022; Ito et al., 2023). Incorporating such markers into clinical protocols strengthens mechanistic interpretation and increases the likelihood of detecting target engagement in early-phase trials.

### 4.4 Informing power calculations

An underappreciated advantage of ALI culture is its incorporation of donor variability. Each insert typically represents cells derived from an individual donor. Consequently, inter-individual variability in viral replication and drug responsiveness can be quantified in vitro.

Viral susceptibility, innate immune responses in epithelium, cytokine production, and antiviral efficacy may vary substantially among donors. Such variability is absent in immortalized cell lines but may provide valuable information regarding expected treatment heterogeneity in human populations (**Table 1**). This is an increasingly important consideration in precision medicine and clinical trial design. Estimation of variance across donors provides valuable information for statistical power calculations in clinical trial design. This feature moves ALI systems beyond mechanistic biology into practical trial planning. For example, RV568, a narrow-spectrum kinase inhibitor, which inhibits corticosteroid-resistant virus-induced inflammation, was tested in RSV-infected ALI culture and human virus challenge model (Cass et al., 2013; Charron et al., 2017). Using the results of RSV-induced IL-8 inhibition in ALI culture, the sample size was calculated to achieve 50% inhibition of IL-8 release in the RSV human challenge clinical trial. Clinical trials lately revealed that RV568 inhibited RSV-induced IL-8 release by 46%. This is probably the first case showing the translation of ALI data for power calculation (Cass et al., 2013).

## 5 Limitations and integration with emerging platforms

Although several antiviral agents have demonstrated concordance between ALI efficacy and clinical activity, examples also exist in which promising antiviral activity observed in preclinical epithelial models did not translate into clinical benefit. Importantly, these discrepancies do not necessarily indicate a failure of the ALI model itself, but rather reflect limitations inherent to any epithelial-only system. Factors such as inadequate drug exposure at the site of infection, suboptimal treatment timing, host immune responses, patient heterogeneity, and disease mechanisms extending beyond epithelial infection may all contribute to differences between preclinical and clinical outcomes.

One of the major drawbacks is that the system does not replicate systemic immune responses, vascular components, or adaptive immunity. Circulating immune cell recruitment and whole-organism pharmacokinetics cannot be fully captured. Regarding the virus clearance, the virus remains detectable at later time points, especially when virus PCR is used as the endpoint (Brookes et al., 2018). In human infection, viral clearance is a complex process involving both epithelial-intrinsic antiviral mechanisms and coordinated innate and adaptive immune responses, including recruitment of immune cells, production of virus-specific antibodies, and T-cell-mediated elimination of infected cells. In fact, many of RSV virus challenge studies, the virus was completely or nearly cleared within one week (Mackman et al., 2015; DeVincenzo et al., 2020; Ahmad et al., 2022). These systemic and cellular immune components are not represented in conventional ALI epithelial cultures although some attempts were made (Yonker et al., 2017; Luukkainen et al., 2018; Iriondo et al. 2025). Thus, viral decline or loss of detectable virus observed in ALI cultures should not be interpreted as equivalent to complete viral clearance in vivo. Emerging approaches integrating immune cell co-cultures, organ-on-chip systems, and microfluidic platforms may further enhance physiological relevance (Nawroth et al., 2020; Wang et al., 2021; Ringquist et al., 2025). Nonetheless, an additional advantage of ALI models is that they are technically easier to obtain and culture compared with more complex models. Secondary, co-morbid conditions and microbiome interactions are only partially represented. Furthermore, because of the closed system in a round shape well, treatment won’t be cleared until mechanical washout is conducted, and the cilia movement direction is not one-way or synchronised; consequently it does not mimic mucociliary clearance in humans.

Thirdly, particularly, this system is better suited for evaluating inhaled antiviral strategies, but probably not perfect for oral anti-viral drugs. For inhale medicine, drug concentrations measured in the epithelial lining fluid or airway lumen can be directly related to antiviral efficacy endpoints, such as viral load reduction, viral clearance kinetics, and biomarker modulation. This allows estimation of clinically relevant target concentrations and supports translational pharmacokinetic–pharmacodynamic (PK–PD) modelling. In contrast, prediction of efficacy for systemically administered antivirals is inherently more complex because antiviral activity depends not only on intrinsic potency but also on multiple pharmacokinetic processes, including absorption, plasma protein binding, tissue distribution, metabolism, and penetration into the airway epithelial compartment. ALI models can still provide valuable information regarding antiviral potency and epithelial responses for systemically administered compounds; For example, RSV fusion inhibitors such as presatovir showed potent antiviral effects in preclinical systems and human challenge studies but failed to demonstrate consistent clinical benefit in naturally infected patient populations. These examples highlight an important limitation of ALI systems: Therefore, additional *in vivo* pharmacokinetic data and modelling are generally required to accurately predict airway exposure and clinical efficacy after oral treatment. The latest microfluid system offers the connection of lung-on-a-chip to liver-on-a-chip, and drugs can be tested in more complicated conditions.

## 6 Conclusion

**Figure 1:**
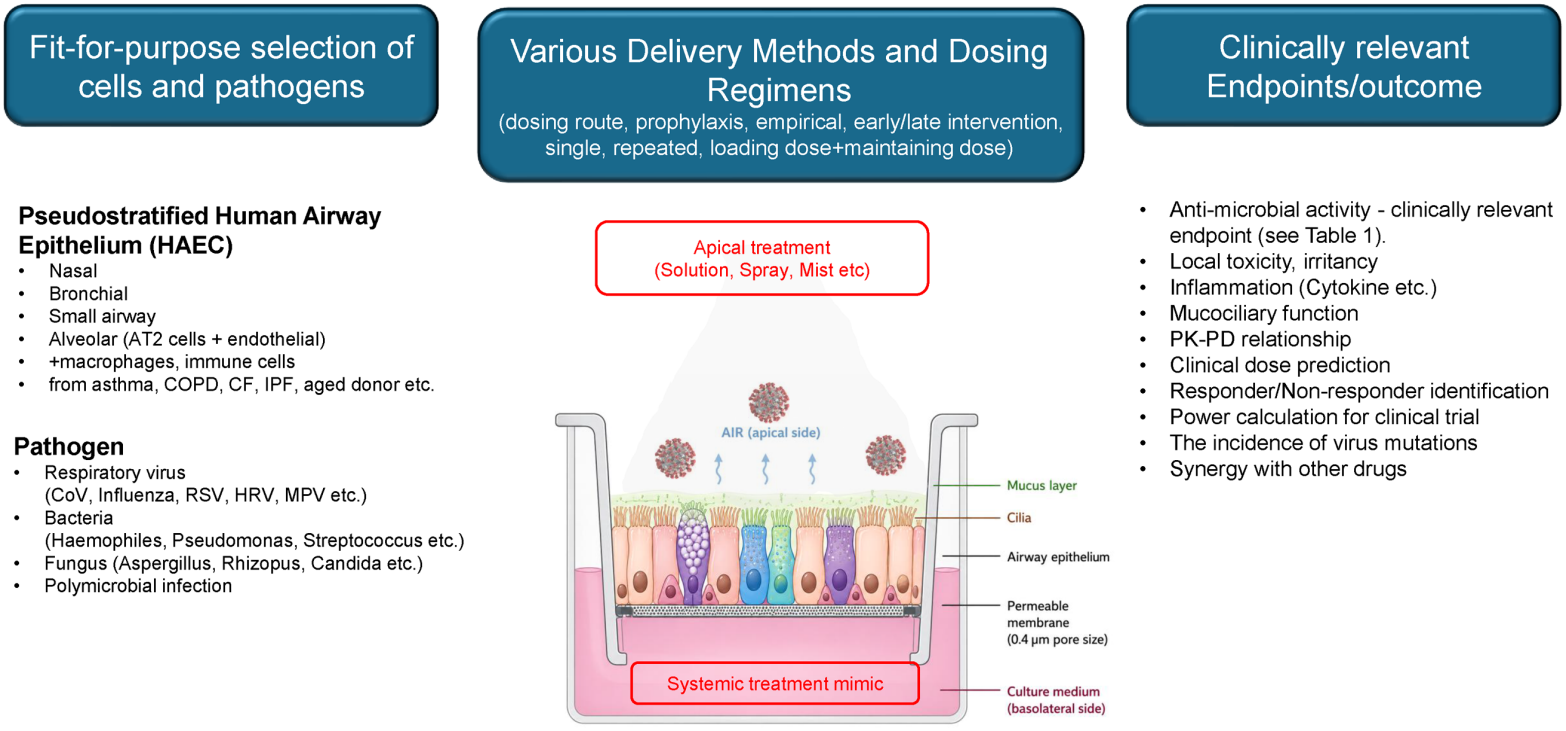
Utility of the ALI culture system for anti-microbial agent development. AT2, alveolar type 2; CF, cystic fibrosis; COPD, chronic obstructive pulmonary disease; CoV, coronavirus; hRV, human rhinovirus; IPF, idiopathic pulmonary fibrosis; MPV, metapneumovirus; PK-PD, pharmacokinetics-pharmacodynamics; RSV, respiratory syncytial virus.

ALI-cultured human airway epithelium has progressed beyond its original role as a descriptive in vitro model and is now widely used as a translational tool for antiviral and anti-infective drug development. Because these cultures generate differentiated and polarized airway tissue with functional host defence pathways, they can reproduce clinically relevant features such as viral replication dynamics, epithelial immune responses, and inter-donor heterogeneity more effectively than conventional submerged monolayer cultures (**Figure 1** and **Table 1**). Experience from clinical development programs of several antiviral compounds further supports the translational relevance of findings generated in ALI systems.

In addition to mechanistic studies, ALI platforms are increasingly incorporated into translational decision-making processes, including estimation of clinically relevant exposure, biomarker assessment, alignment of preclinical and clinical outcome measures, and optimisation of clinical trial design. Consequently, these systems provide an important link between simplified cellular assays and resource-intensive animal or human studies. Their capacity to integrate mechanistic biology with pharmacological, biomarker, and variability-related readouts within a unified experimental framework may help reduce failure during clinical translation. Regulatory agencies and the pharmaceutical industry are also showing growing interest in human-relevant epithelial platforms as supportive tools for early development strategies.

As respiratory antiviral research moves toward more predictive and precision-based approaches, ALI airway models, particularly when integrated with AI-driven analytical platforms in future, are becoming an increasingly important technology for linking experimental discovery with clinical applications.
